# Case Report: Severe Immune-Related Cholestatic Hepatitis and Subsequent Pneumonia After Pembrolizumab Therapy in a Geriatic Patient With Metastic Gastric Cancer

**DOI:** 10.3389/fmed.2021.719236

**Published:** 2021-10-12

**Authors:** Hui Yang, Chenfei Zhou, Fei Yuan, Liting Guo, Liu Yang, Yan Shi, Jun Zhang

**Affiliations:** ^1^Department of Oncology, Ruijin Hospital, Shanghai Jiaotong University School of Medicine, Shanghai, China; ^2^Department of Pathology, Ruijin Hospital, Shanghai Jiaotong University School of Medicine, Shanghai, China

**Keywords:** immune checkpoint inhibitor, multisystem immune-related adverse event, immune-mediated hepatitis, cholestasis, late-onset pneumonia

## Abstract

**Background:** Immune checkpoint inhibitors have provided significant clinical benefits to many patients with advanced cancer; however, severe immune-related adverse events (irAEs) have occurred. Detecting and treating irAEs early could improve patient prognoses. Therefore, clinicians and patients should understand that these irAEs exist, especially those that are rare and serious.

**Case Presentation:** In this report, an 86-year-old male patient, diagnosed with metastatic gastric cancer involving the peritoneum and retroperitoneal lymph nodes was treated with 5-cycle pembrolizumab therapy (100 mg q 2 weeks), achieving a partial response. However, the patient developed Grade 3 cholestatic hepatitis and delayed pneumonia 10 days and 2 months after the final pembrolizumab dose, respectively. After discontinuing the pembrolizumab therapy and excluding obstructive jaundice with imaging studies, the patient received steroid therapy, with a gradual symptom improvement. However, the patient developed delayed pneumonia with type 1 respiratory failure 1-month post-discharge. Several microbiologic tests were negative, and immune-associated pneumonia was suspected, but we could not exclude an opportunistic infection. The patient recovered with steroids and antibiotics and remained in partial remission 5 months after pembrolizumab withdrawal.

**Conclusions:** Cholestatic hepatitis is a rarely reported toxicity of immune checkpoint inhibitors, which should be suspected and addressed once obstructive jaundice is ruled out. In addition, clinicians should be aware that irAEs can occur at any time in patients treated with immune checkpoint inhibitors and that a timely diagnosis should be made.

## Background

Immune checkpoint inhibitors (ICIs) have revolutionized cancer therapy. Studies of metastatic cancer being treated with antibodies against T lymphocyte-associated antigen 4 (CTLA-4) and programmed cell death 1 (PD-1) PD-1/PD-1-ligand 1 (PDL-1) pathways have been expanding and are widely used to treat several solid tumor types. Durable therapeutic responses have been demonstrated, showing anti-tumor immunity in several solid tumors, including melanoma, non-small cell lung cancer (NSCLC), and refractory Hodgkin's lymphoma (1, 2).

However, ICIs can reactivate auto-reactive immune cells and tumor-specific T cells, leading to immune-related adverse events (irAEs) in almost 70% of patients. Most irAEs are mediated by T-cells and CD4 and CD8 T cell infiltrations. B cells, granulocytes, and cytokines have also been implicated (3). This over-stimulated immune reaction leads to autoimmune reactions. These AEs can be temporary or chronic, mild or life-threatening, and involve almost any and sometimes multiple organ systems (4). They are diverse and often markedly different from those observed with traditional cytotoxic chemotherapeutic and radiation cancer treatments (5). Skin, intestine, thyroid, lung, liver, and joints are most frequently involved, and myocarditis, pneumonia, and encephalitis can be fatal. Therefore, understanding the clinical manifestations and differentiation of irAEs, especially rare irAEs, can lead to efficient diagnoses and early treatment strategies to improve prognoses.

This case report describes an elderly patient with metastatic gastric cancer who developed a rare severe immune-related cholestatic hepatitis and subsequent severe late-onset pneumonia after pembrolizumab therapy.

## Case Presentation

**Figure 1:**
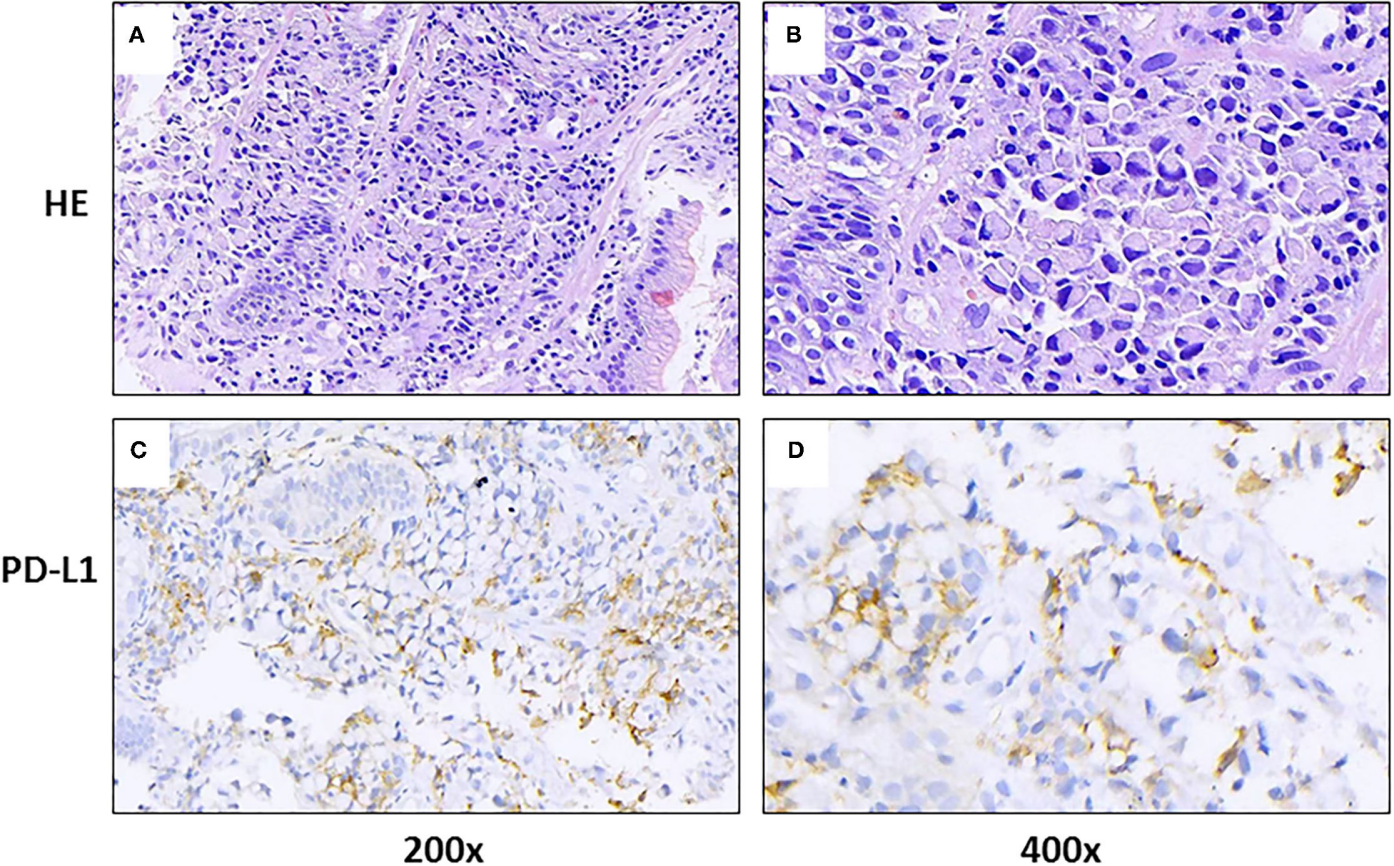
Gastric tissue sections from a patient with metastatic gastric cancer and rare severe immune-related cholestatic hepatitis and subsequent severe late-onset pneumonia after pembrolizumab therapy. Representative photomicrographs show H&E staining **(A,B)** and immunohistochemical staining with programmed death-ligand 1 (PD-L1) **(C,D)**, using a PDL1 antibody (DACO, 22C3).

**Figure 2:**
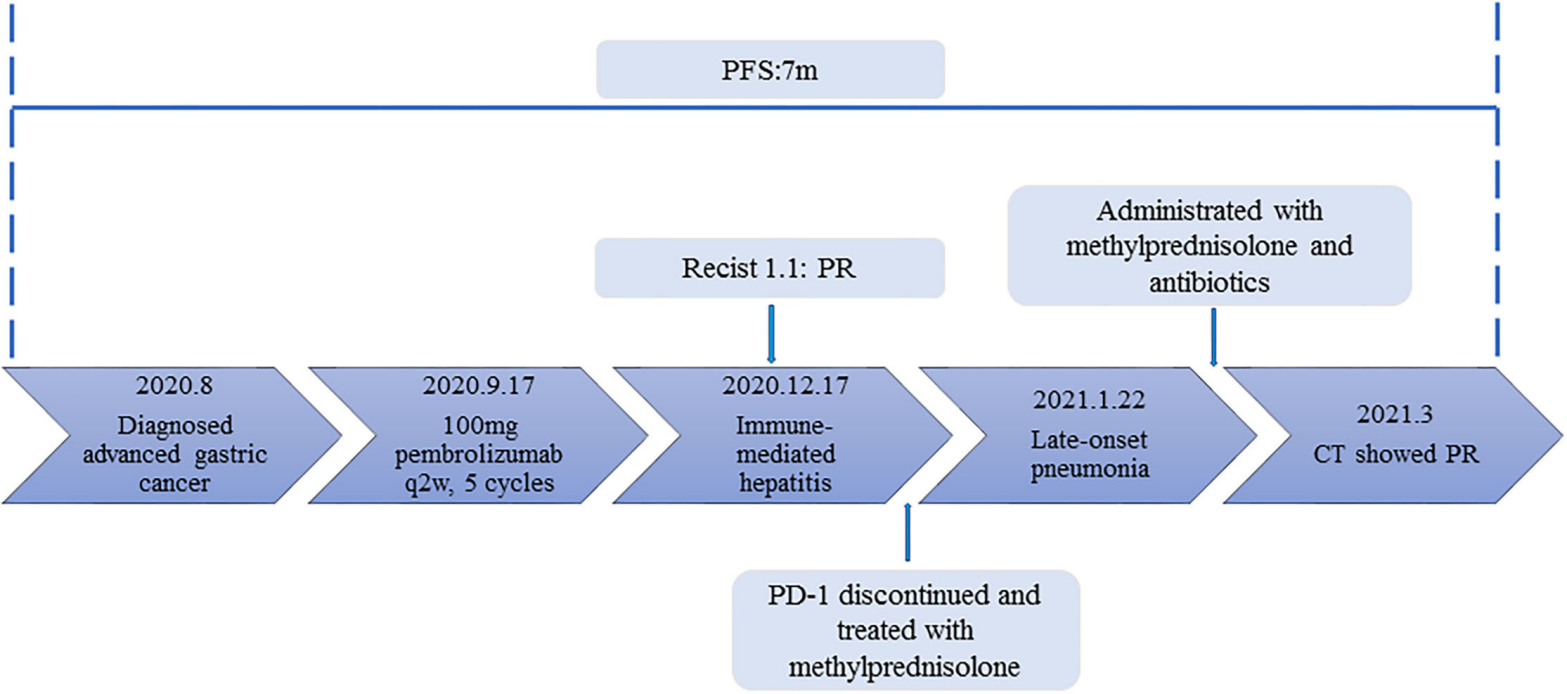
The timeline of diagnosis, treatment and related immune-side effects of this patient since diagnosis.

**Figure 3:**
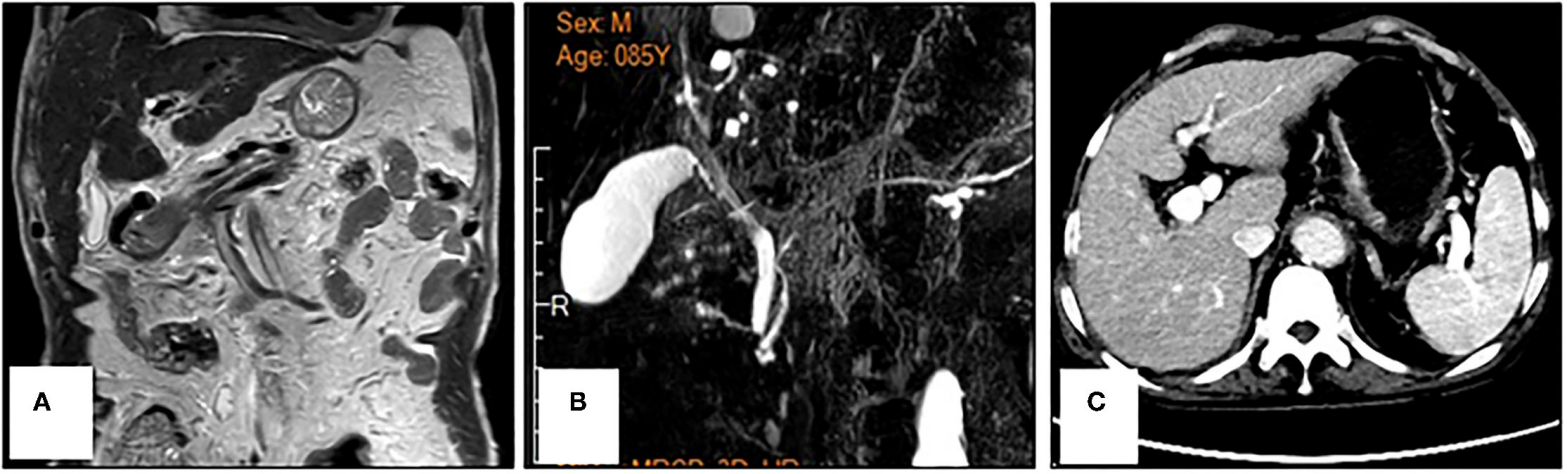
Reprensentative images of magnetic resonance cholangiopancreatography **(A,B)** and abdominal computed tomography **(C)** in a patient with metastatic gastric cancer and rare severe immune-related cholestatic hepatitis and subsequent severe late-onset pneumonia after pembrolizumab therapy. No obvious biliary obstruction was observed.

**Figure 4:**
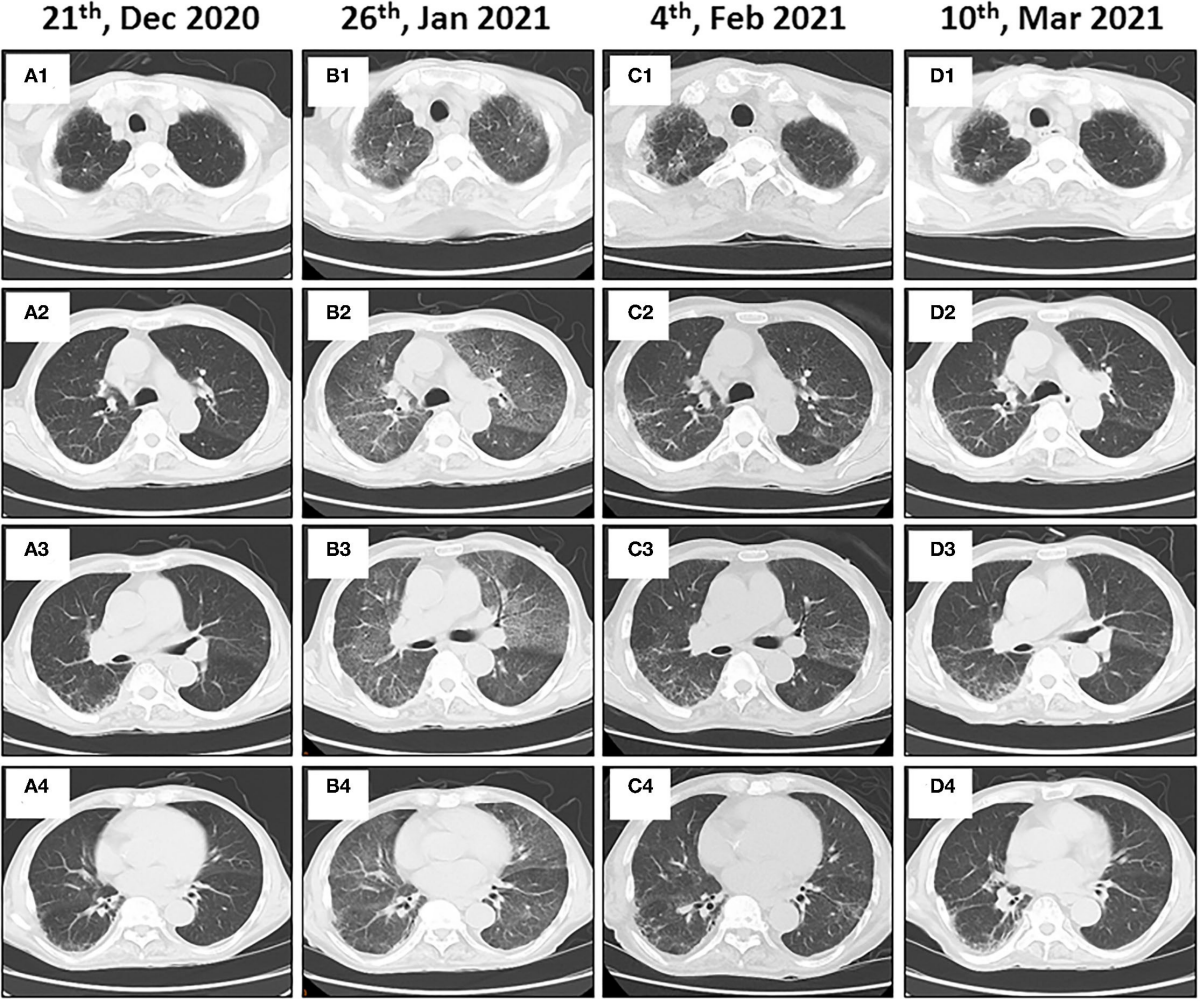
Immune-related pneumonitis in a patient with metastatic gastric cancer and rare severe immune-related cholestatic hepatitis and subsequent severe late-onset pneumonia after pembrolizumab therapy. Baseline chest computed tomography (CT) images show the lungs before starting immunotherapy **(A1–A4)**. Follow-up axial CT images obtained 4 months after administering the pembrolizumab therapy showing non-specific interstitial pneumonia **(B1–B4)**. Immunotherapy was halted, and steroid therapy was administered. Follow-up chest CT images show near complete resolution of pneumonitis **(C1–C4)**. Chest CT images obtained after the steroid therapy was discontinued for 5 weeks **(D1–D4)**.

An 86-year-old male patient presented to Ruijin Hospital, Shanghai Jiao Tong University School of Medicine, because of the increase of CA-199 level to 282.9 U/mL in regular physical examination, without nausea, vomiting, acid reflux, belching, or other symptoms with an Eastern Cooperative Oncology Group (ECOG) performance status (PS) of 1. First, abdominal contrast-enhanced CT was performed, and showed that the gastric wall of cardia was thickened with abnormal enhancement in arterial phase, the outer margin of gastric wall was rough, and multiple nodules were scattered in hepatogastric ligament, retroperitoneum, peritoneum, omentum majus and mesangium. The malignant tumor infiltrated the entire layer of gastric cardia, and multiple round lymph node were highly suspicious for metastasis in the abdominal cavity. Second, the upper GI endoscopy subsequently identified proliferative ulcers in the region of the cardia, and signet ring cell carcinoma was confirmed by pathology. PET/CT was also demonstrating obvious cardia thickening with significant FDG uptake (SUvmax 14.3), as well as increased metabolic activities in the enlarged abdominal lymph nodes (SUvmax 4.9 to 6.8), and nodular thickening of the right retroperitoneum (SUvmax 3.8). Immunohistochemical stainings of the gastric tissue demonstrated positive expressions of MLH1, PMS2, MSH2, and MSH6, PD-L1 combined positive score (CPS) of 20, Ki-67 expression level of 90%, and negative HER-2 expression (Figure 1). He was diagnosed with metastatic gastric cancer with multiple abdominal cavity lymph node and peritoneal metastases (HER2 negative, pMMR, and PD-L1 CPS 20). Chemotherapy was recommended as the first-line treatment, but the patient and his family refused due to his old age and frailty. Given his high PD-L1 CPS and the significant overall survival (OS) improvement in patients with CPS ≥ 10 treated with pembrolizumab compared to those with chemotherapy in the KEYNOTE-062 trail (6), he was recommended and administered with 100 mg pembrolizumab as the first-line chemotherapy every 2 weeks for 5 cycles (Figure 2). Ten days after the last dose, the patient presented for icteric sclera and skin and generalized pruritus. He was administrated with the ursodeoxycholic acid (250 mg, bid) in other hospitals for 1 week before admission to our hospital, but the above symptoms did not improve. After addimission, the patient remained hemodynamically stable and the abdominal exam findings were benign. On serum biochemical testing, a marked increase in total and direct bilirubin and slight increase in γ-glutamyl transferase were identified, the level of alkaline phosphatase (ALP; range, 30–120 IU/L) also increased, but alanine aminotransferase (ALT; range, <50 IU/L) and aspartate aminotransferase (AST; range <50 IU/L) were within normal limits (Table 1). We assessed the tumor status, and a partial response (PR) was assigned according to RECIST criteria. Neither magnetic resonance cholangiopancreatography (MRCP) nor abdominal computed tomography (CT) showed significant biliary obstruction or bile duct dilatation (Figure 3). Tumor progression, and biliary inflammation and obstruction were ruled out. We suggested liver biopsy for differential diagnosis, but the patient and his family refused due to his old age and poor physical condition on admission. Given the patient's PD-1 treatment history, we therefore diagnosed him with immune-related cholestatic hepatitis was diagnosed, and ICI treatments were suspended. The patient received pulse therapy with 80 mg methylprednisolone. Simultaneously, ursodesossicolic acid was administrated to this patient daily. Icterus resolved after 3 days, and the pruritus significantly improved. Total bilirubin decreased from 171.4 to 139.3 μmol/L and direct bilirubin decreased from 114.3 to 88.4 μmol/L after 3-day high-dose steroids treatment. Therefore, oral dexamethasone replaced the methylprednisolone with a tapered dose (dexamethasone 7.5 mg for 4 days, 6 mg for 4 days, and 3 mg for 4 days). Total and direct bilirubins continued to decreasedecreasing, and he was discharged from hospital on day 36 (see Table 1). One month later, the patient developed asthma with a low-grade fever that increased slightly (37.8–38.5°C) in the hospital. Oxygen saturation was low at 78–90%, and a decreased white cell count (1.56 × 10^9^/L; range [4–10 × 10^9^/L]), high levels of C-reactive protein (72 mg/L; range, <10 mg/L), and procalcitonin (0.1 ng/ml; range, <0.5 ng/mL) were found on blood tests. The 1, 3-β-D-glucan detection (G test) and galactomannan antigen detection (GM test) were performed using blood samples, while bacterial and fungal cultures were performed on sputum and blood. In addition, SARS-CoV-2 nucleic acid test through throat swab specimens and tuberculosis -related sputum and blood tests were also performed. All tests were negative and the antibiotic, Cefoperazone Sodium and Sulbactam Sodium^(sulperazone)^, was administered with granulocyte-colony stimulating factor to promote leucocyte production; however, no significant symptomatic improvement was seen. Another chest CT showed interstitial changes, and interstitial pneumonia was considered probable (Figure 4). He was diagnosed with type 1 respiratory failure based on the report of arterial blood gas examination and treated with high-flow nasal oxygen. A multi-disciplinary consultation was completed, and since the patient had received a PD-1 inhibitor, a consensus considered immune-related pneumonia to be likely. However, because the patient was elderly and treated with glucocorticoids for a considerable time, opportunistic infections could not be excluded. Therefore, the patient was given pulse therapy with 80 mg methylprednisolone and levofloxacin, meropenem, and sulfamethoxazole/trimethoprim. No significant improvements were seen after 3 days, and the patient was transferred to ICU for high-flow oxygen and intravenous immunoglobulin (25 g/day) therapy. An antifungal drug, caspofungin^(coses)^, was also added. Symptoms improved 6 days later, and resolution of the interstitial lung changes was seen on CT (Figure 4). The patient was discharged, and antibiotics were discontinued. At a 1 month recheck, the patient was in good general health with an ECOG PS of 1, and bilirubin levels were within normal limits (Table 1). Enhanced CT of the abdominopelvic cavity showed that PR was maintained (Figure 2) with progression-free survival (PFS) of 7 months.

**Table 1:** Specific hematologic and serum biochemical data in a patient with metastatic gastric cancer and rare severe immune-related cholestatic hepatitis and subsequent severe late-onset pneumonia after pembrolizumab therapy.

**Hospital day**	**Total bilirubin** **(5–21 μmol/L)**	**Direct bilirubin** **(<3.4 μmol/L)**	**AST** **(<50 IU/L)**	**ALT** **(<50 IU/L)**
1	12.8	4.9	40	43
**11**	**171.4**	**114.3**	49	54
16	139.3	88.4	38	41
18	115.1	69.9	35	35
21	102.5	60.5	37	37
23	101.3	57.9	46	42
27	73.7	41.3	50	43
30	68.7	38.7	62	41
33	55	30.7	57	36
46	43.5	21.5	42	49
49	29.5	15.5	36	33
90	26.2	15.7	38	48

*AST, aspartate aminotransferase; ALT, alanine aminotransferase. The bold values indicates the peak level of total bilirubin and direct bilirubin in this patient*.

## Discussion and Conclusions

Most irAEs occur in the first 4 months after ICI therapy (7), but delayed toxicities can occur months after treatments are discontinued. The overall incidence for any irAE is 50–100%, and most are grades 1–2, but grades 3–4 have been shown in 7–12% of patients receiving single anti-PD-1 drugs (7, 8). Immune-mediated hepatitis (IMH) has an incidence of 1–4% for liver injury and 60% for cholestatic injury (9). However, liver enzymes are rarely increased, occurring in <5% of ICI monotherapy clinical trials, and severe hepatitis is particularly rare (10). In this case, the injury was primarily cholestatic with a nearly 8-fold increase in bilirubin and a grade 3 classification according to the Common Terminology Criteria for Adverse Events, Version 5.

Little is known regarding the etiology, diagnosis, and treatment of ICI hepatitis. Most cases have lymphoblastic or granulomatous infiltrations, although liver pathology can be diverse. Plasma cell infiltrations, which are common in autoimmune hepatitis, appear to be less common in ICI hepatitis, suggesting that ICI hepatitis has a unique underlying etiology (11, 12). Regulatory T cells (Tregs) are crucial for maintaining immune tolerance. Considering the high expression of immune checkpoint molecules such as CTLA4 and PD-1 on Treg cells, ICIs may target Tregs. Hence, dysfunction of Tregs may contribute to the development of iRAEs in part (13). Cancer patients are at higher risk for liver damage from liver metastases, biliary tract compression, opportunistic infections, and drug reactions (14). For these reasons, ICI-treated patients with elevated serum liver enzymes should be evaluated for ICI hepatitis, including thorough laboratory and imaging examinations to rule out other causes of liver injury.

In this case, immune-related cholestatic hepatitis was diagnosed based on the exclusion of other causes of cholestatic liver diseases by laboratory tests and imaging examinations. The common differential diagnoses of cholestatic hepatitis include hemolytic jaundice, obstructive jaundice, hepatocyte jaundice, primary biliary cholangitis (PBC), and primary sclerosing cholangitis (PSC), as well as immune-related cholestatic hepatitis. We first ruled out hemolytic jaundice and hepatocellular jaundice through laboratory tests; subsequently, obstructive jaundice and PSC were excluded by MRCP and abdominal CT/MRI. PBC and PSC are two major diseases leading to cholestatic liver injury in adults (15). PBC is an autoimmune disease, and the specific antibody including anti-mitochondrial antibodies (AMA-M2) and antinuclear antibodies (ANA: sp100 and gp210) are positive in PBC. The lack of autoimmune antibody testing was our negligence. Furthermore, liver histology is a valuable tool to classify PBC (16). However, the patient and his family refused liver biopsy due to his old age and poor physical condition on admission. In addition, we did not detect autoimmune antibodies, mainly considering that the patient had an acute onset, had no history of autoimmune diseases, and the patient had been treated with ursodeoxycholic acid in other hospitals for 1 week before admission, and the symptoms did not improve, which were not in line with the common clinical manifestations of PBC. Given the patient's PD-1 treatment history, we therefore diagnosed the patient with immune-related cholestatic hepatitis, and timely gave him high -dose steroids to improve his symptoms. PSC primarily affects the large bile ducts, and the gold diagnosis standard of PSC is endoscopic retrograde cholangio-pancreaticography (ERCP) and magnetic resonance cholangio-pancreaticography (MRCP) by far (17). MRCP showed no abnormal changes in the large bile ducts, specifically the extrahepatic ducts, so PSC was also excluded. However, we have to admit that the lack of liver biopsy and autoimmune antibody detection were the main limitations during our diagnosis.

The current guidelines for ICI hepatitis are primarily based on NCCN guidelines for management of immunotherapy -related toxicity, and steroid hormones are the primary medication for grade 2 or higher immune-associated liver injury (18, 19). Most ICI-IMH cases are sensitive to steroids, and complete healing can occur in 6–12 weeks (20). In this study, total bilirubin began to decrease 3 days after the methylprednisolone was initiated and decreased to normal after 2 months of therapy.

ICI pneumonia has occurred in ~1–5% of previously reported studies and might be more frequent in NSCLC patients (21). It can lead to severe morbidity, treatment interruptions, and possibly death. The occurrence of ICI pneumonia can vary widely, ranging from 9 days to 19 months after treatment initiation, with a median onset of 2.8 months (22, 23). ICI pneumonia has been described in patients receiving PD-1 inhibitors for advanced NSCLC 4 weeks after prednisone was discontinued with good responses to systemic corticosteroids (24). This phenomenon was attributed to the lasting effects of ICIs, producing prolonged tumor responses even after drug withdrawal.

Previous studies have shown that cancer patients treated with ICI monotherapy developed multiple systemic irAEs, including pneumonic thyroiditis, hepatitis thyroiditis, dermatitis pneumonia, and inflammatory thyrodermatitis (25). In our case, the patient developed a rare form of hepatitis and pneumonia. Usually, multisystem irAEs arise from common combinations of single irAEs with shared pathobiologic characteristics (26, 27); however, the specific mechanisms remain unclear and need further studies. Interestingly, patients with single or multisystem irAEs demonstrated improved OS compared with patients with no irAEs (25). The patient of our report has been well-controlled to date.

In summary, clinicians should be aware that irAEs can occur at any time in ICI-treated patients, and timely differential diagnoses should be made. After excluding other inflammatory conditions, steroid therapy should be given. If microbial infections cannot be completely ruled out, antimicrobials should be empirically given with steroids.

## Data Availability Statement

The original contributions presented in the study are included in the article/supplementary material, further inquiries can be directed to the corresponding author/s.

## Ethics Statement

Written informed consent was obtained from the individual(s) for the publication of any potentially identifiable images or data included in this article.

## Author Contributions

HY, CZ, and YS initiated and wrote the manuscript. FY provided pathological materials. HY, CZ, LG, LY, and YS treated this patient and collected all the relevant clinical materials. YS and JZ revised the manuscript. All authors read and approved the final manuscript.

## Conflict of Interest

The authors declare that the research was conducted in the absence of any commercial or financial relationships that could be construed as a potential conflict of interest.

## Publisher's Note

All claims expressed in this article are solely those of the authors and do not necessarily represent those of their affiliated organizations, or those of the publisher, the editors and the reviewers. Any product that may be evaluated in this article, or claim that may be made by its manufacturer, is not guaranteed or endorsed by the publisher.

## Abbreviations

ICI: immune checkpoint inhibitor
CTLA-4: T lymphocyte-associated antigen 4
PD-1: programmed cell death 1
PD-L1: programmed cell death 1 ligand
irAEs: immune-related adverse events
IMH: immune-mediated hepatitis
ECOG: Eastern Cooperative Oncology Group
PS: performance status
ALT: alanine aminotransferase
AST: aspartate aminotransferase
MRCP: magnetic resonance cholangiopancreatography
CT: computed tomography
PR: partial response
PFS: progression-free survival
NSCLC: non-small cell lung cancer.
